# Modulatory effect of iron chelators on adenosine deaminase activity and gene expression in *Trichomonas vaginalis*

**DOI:** 10.1590/0074-02760150076

**Published:** 2015-11

**Authors:** Muriel Primon-Barros, Graziela Vargas Rigo, Amanda Piccoli Frasson, Odelta dos Santos, Lisiane Smiderle, Silvana Almeida, Alexandre José Macedo, Tiana Tasca

**Affiliations:** 1 Universidade Federal do Rio Grande do Sul, Faculdade de Farmácia, Laboratório de Pesquisa em Parasitologia, Porto Alegre, RS, Brasil; 2 Universidade Federal de Ciências da Saúde de Porto Alegre, Laboratório de Biologia Molecular, Porto Alegre, RS, Brasil; 3 Universidade Federal do Rio Grande do Sul, Faculdade de Farmácia, Laboratório de Tecnologia Bioquímica, Porto Alegre, RS, Brasil; 4 Universidade Federal do Rio Grande do Sul, Centro de Biotecnologia, Porto Alegre, RS, Brasil

**Keywords:** *Trichomonas vaginalis*, adenosine deaminase, iron chelators, purinergic system

## Abstract

*Trichomonas vaginalis* is a flagellate protozoan that parasitises the urogenital human tract and causes trichomoniasis. During the infection, the acquisition of nutrients, such as iron and purine and pyrimidine nucleosides, is essential for the survival of the parasite. The enzymes for purinergic signalling, including adenosine deaminase (ADA), which degrades adenosine to inosine, have been characterised in *T. vaginalis*. In the evaluation of the ADA profile in different *T. vaginalis* isolates treated with different iron sources or with limited iron availability, a decrease in activity and an increase in ADA gene expression after iron limitation by 2,2-bipyridyl and ferrozine chelators were observed. This supported the hypothesis that iron can modulate the activity of the enzymes involved in purinergic signalling. Under bovine serum limitation conditions, no significant differences were observed. The results obtained in this study allow for the assessment of important aspects of ADA and contribute to a better understanding of the purinergic system in *T. vaginalis* and the role of iron in establishing infection and parasite survival.

*Trichomonas vaginalis *is a flagellate that parasitises the human urogenital tract and causes trichomoniasis, the most common, nonviral sexually transmitted disease (STD) in the world. The worldwide prevalence of this infection is 187 million people (WHO 2012). In women, the clinical characteristics of the infection are associated with vaginitis and cervicitis (Petrin et al. 1998). In men, trichomoniasis may occur as urethritis or prostatitis (Sutcliffe 2010). Complications from this STD are associated with cervical cancer (Viikki et al. 2000), infertility (Goldstein et al. 1993), adverse pregnancy outcomes (Klebanoff et al. 2001), preterm delivery, low birth weight (Cotch et al. 1991, 1997), aggressive prostate cancers (Sutcliffe 2010) and increased risk of human immunodeficiency virus contraction and transmission (Sorvillo et al. 2001). The establishment and maintenance of a *T. vaginalis *infection depends on the successful colonisation of the mucous membranes, adherence, cytotoxicity against vaginal epithelial cells or prostatic cells and the ability to evade the immune system and acquire nutrients (Lehker & Sweeney 1999, Figueroa-Angulo et al. 2012).

In addition to iron, purine and pyrimidine nucleosides are among the essential nutrients for *T. vaginalis *survival. In vivo, these nutrients are acquired from vaginal microorganisms and host cells (Petrin et al. 1998). In vitro, culture media are typically supplemented with bovine or horse serum, which provides these nutrients (Linstead 1989). Adenosine is the primary precursor of all purine nucleotides in*T. vaginalis, *but because it lacks *de novo *purine and pyrimidine synthesis, it depends on the salvage pathway for their acquisition (Munagala & Wang 2003). Iron plays a crucial role in trichomoniasis pathogenesis (Lehker & Alderete 1992, Garcia et al. 2003).*T. vaginalis *possesses multiple iron uptake systems (Ardalan et al. 2009, Torres-Romero & Arroyo 2009). Studies have demonstrated that iron influences the expression of multiple genes that are implicated in virulence factors (Figueroa-Angulo et al. 2012). Hydrolysis of extracellular nucleotides, such as extracellular adenosine triphosphate (ATP), adenosine diphosphate (ADP) and adenosine monophosphate (AMP), is also modulated by iron (Tasca et al. 2005, de Jesus et al. 2006).

ATP and adenosine are involved in various biological and pathological processes. ATP is released during stress, anoxia or injury and acts as a signalling molecule for the immune system (Robson et al. 2006). During purinergic signalling, nucleotide metabolism is a crucial factor that modulates inflammation and specific immune responses (Sansom et al. 2008). This nucleotide metabolism is performed by an enzymatic chain consisting of ectonucleoside triphosphate diphosphohydrolase (ecto-NTPDase family), ecto-5'-nucleotidase and adenosine deaminase [(ADA); EC 3.5.4.4). Ecto-NTPDase hydrolyses nucleotide di and triphosphates to form monophosphate nucleotide. Ecto-5'-nucleotidase breaks down monophosphate nucleotides, which, in turn, leads to the activation of the P1 receptor or deamination by ADA (Tasca et al. 2004, Weizenmann et al. 2011). ADA degrades either adenosine or 2'-deoxyadenosine to produce inosine or 2'-deoxyinosine, respectively (Franco et al. 1997). ADA has been described in mammalian cells and tissues, haematophagous insects, mollusks, bacteria and parasites, such as *Plasmodium lophurae*,*Plasmodium falciparum*, *Trypanosoma evansi*,*Trichinella spiralis*, *Fasciola gigantica*,*Hyalomma dromedarii*, as well as *T. vaginalis*(Ribeiro et al. 2001, Downie et al. 2008, Silva et al. 2011, Weizenmann et al. 2011, Miller et al. 2012).

The presence of ADA in *T. vaginalis *was first mentioned in a study on the characterisation and expression of S-adenosylhomocysteine, in which ADA activity was observed to be very low or even absent (Minotto et al. 1998). Subsequently, ADA activity was identified in *T. vaginalis*lysates. Recently, our research group characterised the kinetics and demonstrated the gene expression of ADA in intact *T. vaginalis*trophozoites (Munagala & Wang 2003,Weizenmann et al. 2011). The presence of ADA, taken together with the presence of NTPDase and ecto-5'-nucleotidase in *T. vaginalis,* suggests a possible modulation of the concentrations of nucleotides/nucleosides during an inflammatory and immune response because extracellular ATP is a potent proinflammatory molecule. On the other hand, adenosine and inosine are antiinflammatory agents that regulate inflammation outcomes. Considering the questions regarding the role of ADA in this parasite's survival, this study investigated the effect of essential nutrients, bovine serum and iron on enzyme activity and gene expression.

## SUBJECTS, MATERIALS AND METHODS

*T. vaginalis *culture and parasite suspension preparation - In this study the *T. vaginalis *isolates 30236 and 30238 from the American Type Culture Collection (ATCC) and the fresh clinical isolates TV-LACM1, TV-LACM2 (from female patients), TV-LACH1 and TV-LACH2 (from male patients), from the Clinical Laboratory, Federal University of Rio Grande do Sul (UFRGS), Brazil (approved by UFRGS Ethical Committee, protocol 18923) were used. The parasites were cultured axenically in trypticase-yeast extract-maltose (TYM) medium (Diamond 1957), pH 6.0, supplemented with 10% (v/v) heat-inactivated bovine serum (HIBS) and incubated at 37ºC. Organisms in the logarithmic phase of growth and exhibiting normal morphology were used in the experiments with microscopic evaluations performed before and after motility and trypan blue (0.2%) viability exclusion assays. Parasites were then harvested by centrifugation and washed three times with phosphate-buffered saline (PBS).To remove zinc and other divalent cations that are capable of interacting with amino acid (aa) residues and inducing the inhibition of ADA activity (Cooper et al. 1997), 2.0 mM ethylenediamine tetraacetic acid and 2.0 mM ethylene glycol tetraacetic acid were added. The final pellet was resuspended and used in subsequent assays.

*Serum limitation condition* - To investigate the influence of serum limitation, *T. vaginalis *isolates were incubated with an initial inoculum of 2.0 x 10^5 ^trophozoites mL^-1^ in TYM medium supplemented with 1% (v/v) HIBS as the serum limitation condition or TYM supplemented with 10% (v/v) HIBS as the control (Frasson et al. 2012). After 24 h of incubation, an ADA assay was carried out. Experiments were performed in triplicate with at least three independent experiments (using different cultures).

*Treatments with iron* - To evaluate the effect of iron on ADA enzyme activity, initially 2.0 x 10^5 ^trophozoites mL^-1^ were inoculated in TYM-serum containing 50 μM of the iron chelator 2,2-bipyridyl (2,2-DP) (Sigma Chemical Co, USA) at 37ºC for 24 h to obtain low-iron-grown trichomonads (Lehker et al. 1991). These organisms were then suspended in TYM-serum supplemented with 50 μM 2,2-DP for an additional 24 h incubation before the experiments. The iron chelator-treated parasites were then washed once and resuspended in TYM-serum medium containing 200 μM ferrous sulphate (FS) (Sigma; high-iron condition), 25 μM haemoglobin (HB) (Sigma) or 25 μM haemin (HM) (Sigma) to obtain iron-replete organisms. The parasites were then incubated for an additional 24 h at 37ºC in the presence of 50 μM 2,2-DP as the low-iron condition. Untreated parasites were used as control. All experiments with the isolates grown under different conditions were performed at least three times in triplicate with different trophozoite suspensions.

*Treatment with ferrozine chelator* - To confirm the iron limitation effect on ADA activity, treatment with a ferrozine chelator was performed by inoculating 2.0 x 10^5 ^trophozoites mL^-1^ in TYM-serum containing 125 μM ferrozine (Sigma) at 37ºC for 24 h. The TYM medium was then replaced by ferrozine for an additional 24 h incubation period (Lehker & Sweeney 1999). Untreated parasites were used as control. The experiments were performed at least three times in triplicate.

*ADA assay* - An ADA enzyme assay was performed by adding a parasite suspension in a reaction mixture containing 50 mM sodium phosphate buffer (pH 7.5) with a protein concentration of 400 μg mL^-1 ^to a final volume of 200 μL. The samples were then pre-incubated for 10 min at 37ºC. The reaction was initiated with the addition of the substrate adenosine (3.0 mM) and stopped after 20 min in order to add the samples to 500 μL of the phenol nitroprusside reagent (50.4 mg of phenol and 0.4 mg of sodium nitroprusside mL^-1^). The controls that had the enzyme preparation added after the reaction had been terminated were used to correct for the nonenzymatic deamination of the substrate. Then, 500 μL of alkaline hypochlorite reagent (sodium hypochlorite to 0.125% available chlorine, in 0.6 M NaOH) were added to the reaction mixtures. Samples were incubated at 37ºC for 15 min. The colorimetric assay was carried out at 635 nm to measure the ammonia produced by the enzymatic reaction. ADA activity was expressed as nmol NH_3_ min^-1^mg^-1^ protein (Giusti 1974). All the assays were performed in triplicate with at least three different experiments. Protein quantification was performed in triplicate using bovine serum albumin as the standard for the parasite suspensions (Bradford 1976).

*Quantitative reverse transcription-polymerase chain reaction (qRT-PCR*) - To evaluate the iron limitation effect on the gene expression of *T. vaginalis *ADA, a qRT-PCR assay was performed. After treatment with 2,2-DP and ferrozine, *T. vaginalis*trophozoites were centrifuged and washed three times with PBS buffer (pH 7.2) for total RNA extraction using TRIzol^®^ (Invitrogen, USA), following the manufacturer's instructions. Then, RNA was quantified by calculating the ratio between the absorbance values at 260 nm and 280 nm. Using 2 μg of total RNA, cDNA was synthesised using the SuperScript^®^ III First Strand Synthesis System for RT-PCR kit (Invitrogen). The amount of cDNA was quantified and its integrity was confirmed by electrophoresis through a 1% agarose gel containing SYBR^®^Green and visualised with an ultraviolet light. qRT*-*PCRs were performed using 280 nM of each primer, 100 ng of cDNA and Power SYBR^®^Green PCR Master Mix (Invitrogen) in a 14 μL total reaction volume using Step One™ Real Time PCR System (Applied Biosystems) equipment. Parallel reactions performed with the cDNA of untreated parasites were used as the positive controls and without reverse transcriptase as the negative controls. During the exponential phase of the qRT-PCR, the threshold cycle (Ct) and baseline were set according to the Applied Biosystems protocols (Miguel et al. 2010). Data from different samples were interpolated on a line created by running standard curves for each primer set and then normalised against the α-tubulin housekeeping gene. All samples and points of the standard curve were performed in duplicate in two independent experiments and analysed according to Livak and Schmittgen (2001). The real-time primer pair sequences that were used are shown in Table.

**TABLE:** Quantitative reverse transcription-polymerase chain reaction primers design

Gene	Primers sequences (5′→3′)	Annealing temperature (ºC)	GenBank accession (mRNA)
TVAG_430670 (*ada 125*)	F - ACGCCAAGAAGCTCGCCGTC R - GAAGCAATGGAAGCGAAACC	57	XM_001325090
TVAG_416510 (*ada 231*)	F - CACCTCTCATGAACAATGCCCTC R - CGAGAACGATCTTTGCGACG	57	XM_001317196
TVAG_206890 (α-tubulin)	F - GCCAACATGATGGTTAAGTGCGATCCAC R - CAGCTTCTTCCATACCCTCACCGACG	61	XM_001330630

ADA: adenosine deaminase.

*Statistical analysis* - ADA activity data were expressed as the mean ± standard deviation and analysed by a one-way analysis of variance followed by Tukey's multiple range test, with p < 0.05 as significant. The analyses were performed using the Statistical Package for the Social Sciences software v.18.

## RESULTS

The effect of the serum limitation condition (1% HIBS) on ADA activity in *T. vaginalis *trophozoites was evaluated. First, the cellular integrity and viability were examined before and after the enzymatic assays by trypan blue dye exclusion. Motility and viability were not affected under any culture conditions. Next, the experimental condition that used trophozoites that had been previously inoculated with 1% HIBS demonstrated that ADA activity was not affected by this condition (data not shown).

**Fig. 1:**
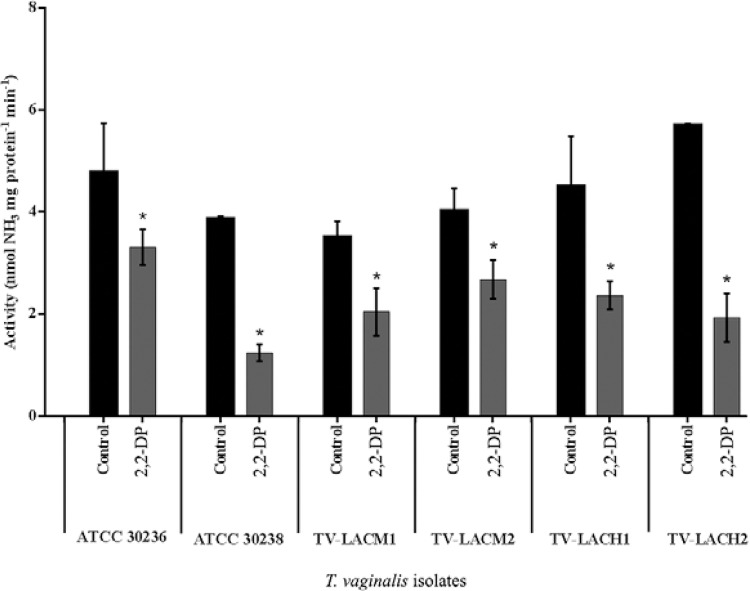
effect of 2,2-bipyridyl (2,2-DP) treatment on *Trichomonas vaginalis *adenosine deaminase activity. There was a significant reduction in enzyme activity when *T. vaginalis*trophozoites were treated with the iron chelator, 2,2-DP, in all isolates tested. Asterisk means significant difference from controls. Bars represent the mean ± standard deviation of three different experiments (parasite suspensions) performed in triplicate. Data were analysed by analysis of variance followed by Tukey's test (p ≤ 0.05). TV-LACH1 and TV-LACH2: fresh clinical isolates from male patients; TV-LACM1 and TV-LACM2: fresh clinical isolates from female patients.

**Fig. 2:**
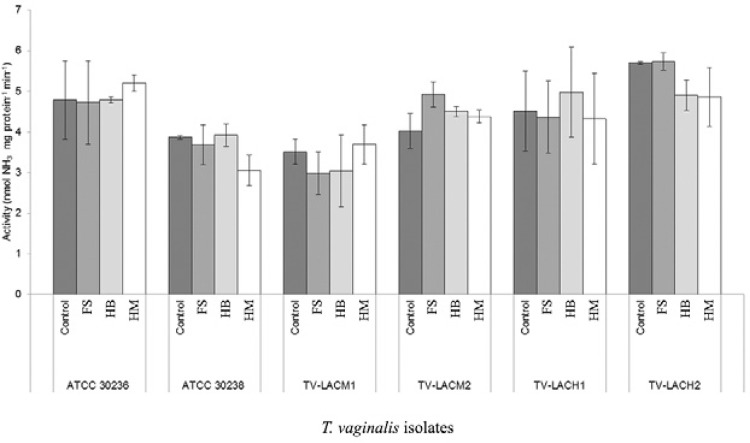
effect of different iron sources treatment, ferrous sulphate (FS), haemoglobin (HB) and haemin (HM), on *Trichomonas vaginalis* adenosine deaminase activity. No significant alteration in enzyme activity was observed. Bars represent the mean ± standard deviation of at least three experiments, each in triplicate, evaluated by analysis of variance followed by Tukey's test (p ≤ 0.05). TV-LACH1 and TV-LACH2: fresh clinical isolates from male patients; TV-LACM1 and TV-LACM2: fresh clinical isolates from female patients.

**Fig. 3:**
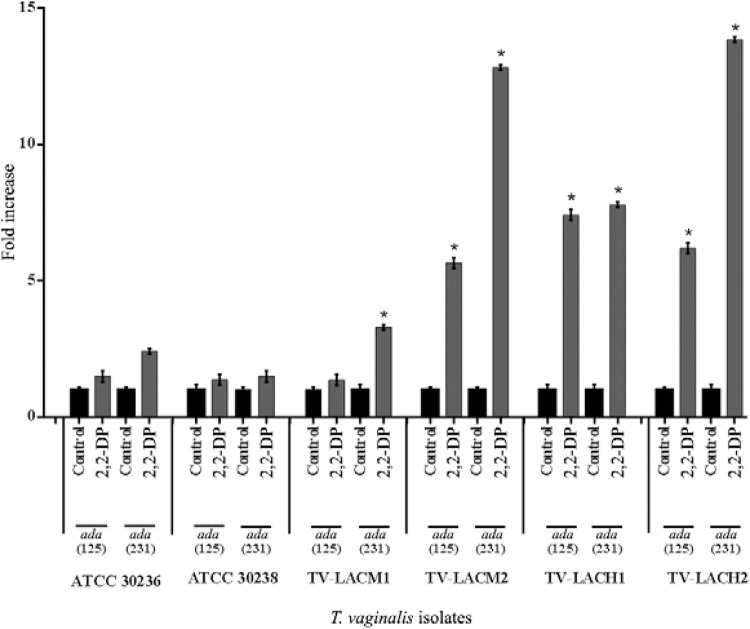
gene expression patterns of adenosine deaminase (ADA) members after iron chelator 2,2-bipyridyl (2,2-DP) treatment in *Trichomonas vaginalis*. There were a increase in gene expression when the parasites were treated with 2,2-DP, providing an iron limitation condition. Data were obtained in duplicate. Data were analysed by analysis of variance followed by Tukey's test (p ≤ 0.05). Asterisk means difference from control TV-LACH1 and TV-LACH2: fresh clinical isolates from male patients; TV-LACM1 and TV-LACM2: fresh clinical isolates from female patients.

**Fig. 4:**
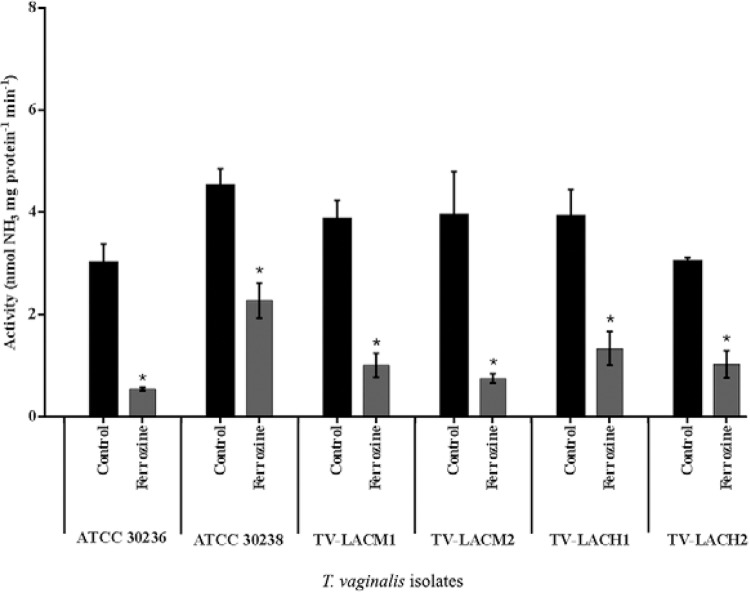
effect of ferrozine treatment on *Trichomonas vaginalis* adenosine deaminase activity. There was a significant reduction in enzyme activity when *T. vaginalis*trophozoites were treated with the iron chelator ferrozine in all isolates tested. Asterisk means significant difference from controls. Bars represent the mean ± standard deviation of three different experiments (parasite suspensions) performed in triplicate. Data were analysed by analysis of variance followed by Tukey's test (p ≤ 0.05). Asterisk means difference from control. TV-LACH1 and TV-LACH2: fresh clinical isolates from male patients; TV-LACM1 and TV-LACM2: fresh clinical isolates from female patients.

**Fig. 5:**
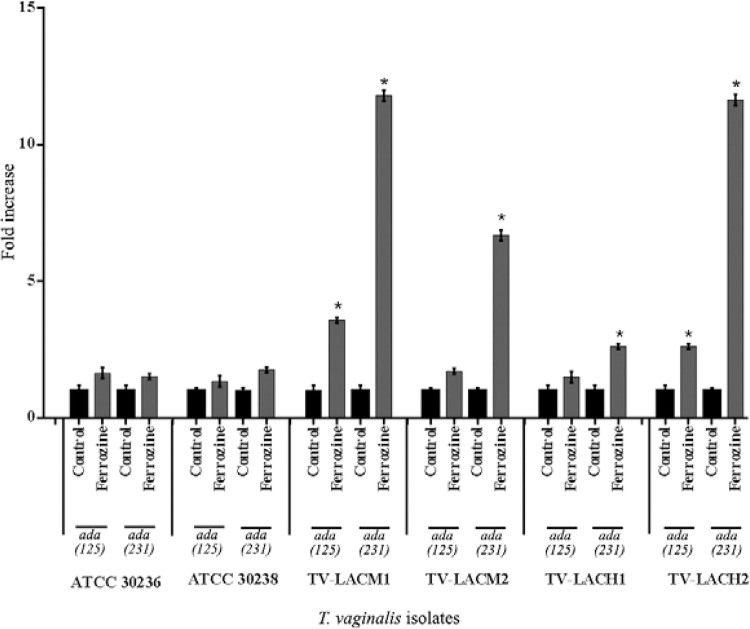
gene expression patterns of adenosine deaminase (ADA) members after treatment with iron chelator ferrozine in *Trichomonas vaginalis*. There was an increase in gene expression when the parasites were treated with ferrozine, providing an iron limitation condition. Data were obtained in duplicate. TV-LACH1 and TV-LACH2: fresh clinical isolates from male patients; TV-LACM1 and TV-LACM2: fresh clinical isolates from female patients.

The effect of the different iron treatments on adenosine deamination in *T. vaginalis *was then evaluated. Cellular integrity was assessed by motility and viability of the trophozoites by trypan blue exclusion before and after all enzymatic assays. Trophozoite integrity was not affected by treatments with iron. The ATCC isolates that had been cultured long-term and the fresh clinical isolates from female or male patients had a decrease in ADA activity after treatment with 2,2-DP (Fig. 1). Decreased enzymatic activity was observed in ATCC 30236 (29%), ATCC 30238 (67.8%), TV-LACM1 (41.8%), TV-LACM2 (27.7%), TV-LACH1 (47.6%) and TV-LACH2 (68.1%). However, trophozoites treated for 24 h with FS, HB and HM did not exhibit significant differences in ADA activity compared with the controls, which indicates that the activity was re-established after treatment with the iron chelator (Fig. 2). To assess whether the decrease in ADA activity that followed 2,2-DP treatment was due to an effect on ADA gene expression in*T. vaginalis*, qPCR experiments were performed. The gene expression of two ADA sequences obtained from the *T. vaginalis*genome was evaluated: TVAG_430670 (*ada 125*) and TVAG_416510 (*ada 231*), both with 733 aa. A comparison of the gene expression in the control trophozoites and that of the *T. vaginalis*treated with the iron chelator 2,2-DP was performed by evaluating the Ct and the baseline obtained during the exponential phase of the qRT-PCR technique and normalising them to the α-tubulin housekeeping gene by calculating the enzyme/α-tubulin ratio. The results indicated that 2,2-DP treatment increased both TVAG_430670 (*ada 125*) and, to a greater extent, TVAG_416510 (*ada 231*) transcript levels in *T. vaginalis*isolates (Fig. 3). TVAG_430670 (*ada 125*) and TVAG_416510 (*ada 231*) genes transcript levels were increased 1.5 and 2.41 times in the ATCC 30236 isolate, 1.37 and 1.50 times in ATCC30238, 1.36 and 3.3 times in TV-LACM1, 5.65 and 12.81 times in TV-LACM2, 7.41 and 7.78 times in TV-LACH1 and 6.19 and 13.83 times in TV-LACH2 (Fig. 3), respectively. To confirm the previous findings, another iron chelator, ferrozine, was tested. Treatment with ferrozine led to the same profile found after 2,2-DP treatment: an 81.7% decrease was observed in the enzymatic activity of the ATCC 30236 isolate. However, for the isolates ATCC 30238, TV-LACM1, TV-LACM2, TV-LACH1 and TV-LACH2, the activity decreased by 51.8%, 72.2%, 80.7%, 65.82% and 66%, respectively (Fig. 4). Additionally, an increase in the transcript levels of TVAG_430670 (*ada 125*) and TVAG_416510 (*ada 231*) genes were observed in ATCC 30236 (1.64 and 1.52 times, respectively), ATCC 30238 (1.34 and 1.76 times, respectively), TV-LACM1 (3.58 and 11.79 times, respectively), TV-LACM2 (1.71 and 6.68 times, respectively), TV-LACH1 (1.50 and 2.62 times, respectively) and TV-LACH2 (2.62 and 11.63 times, respectively) (Fig. 5).

## DISCUSSION

This report assesses the effects of bovine serum limitation and treatment with different iron sources on *T. vaginalis *and highlights the role of iron for these parasites. To investigate the effect of essential nutrients on ADA activity and evaluate a possible heterogeneity among the different isolates, the serum limitation condition assay and treatment with different iron sources or iron limitation conditions were performed using ATCC isolates and fresh clinical isolates from female and male patients. In the serum limitation condition test (1% HIBS), the ADA activity maintenance profile of the control organisms (10% HIBS) was similar to that of all the *T. vaginalis *isolates. These results were not surprising, taking into account that *T. vaginalis *trophozoites do not perform *de novo *purine synthesis and that the serum added to the culture medium is a source of important molecules for parasite survival, especially adenosine. Our findings are in agreement with recent data published by our research group (Frasson et al. 2012), which show a pronounced increase in NTPDase and ecto-5'-nucleotidase activities (increased ATP, ADP and AMP hydrolysis) in parasites in 1% HIBS, compared to the control group. Because adenosine is essential for the parasite, under the serum limitation condition, trophozoites compensate for the levels of adenosine required by not altering ADA enzymatic activity.

Regarding the experiments performed to determine the influence of iron and its role in limiting ADA activity, *T. vaginalis *trophozoites were submitted to different iron treatment conditions. The low-iron treatment condition (iron chelators, 2,2-DP and ferrozine) caused a significant decrease in ADA activity. In contrast, under the other conditions, the enzyme activity was similar to that of the controls. *T. vaginalis *trophozoites were able to re-establish ADA activity after a 24 h exposure period to iron sources (FS, HB and HM). These results are in agreement with the findings by Tasca et al. (2005) in which *T. vaginalis *that had been submitted to high or low-iron treatment conditions did not present changes in NTPDase activity, but ecto-5'-nucleotidase had reduced activity after iron limitation, an effect that did not revert after treatment with a high-iron concentration. In a similar study, de Jesus et al. (2006) investigated the influence of iron in ecto-ATPase and ectophosphatase enzymes by demonstrating a decrease in enzymatic activities with limited iron concentrations and re-established activities after 24 h of treatment with high concentrations of this cation. The observed decrease in the deamination of adenosine to inosine by the lower ADA activity in low-iron concentrations is in agreement with previous findings, considering that adenosine is quickly uptaken to be the primary precursor of the entire purine nucleotide pool in *T. vaginalis *(Harris et al. 1988, Munagala & Wang 2003). In this context, iron can act as a modulator of the enzymatic activities that hydrolyse purine nucleotides and nucleosides.

The qRT-PCR results demonstrated that in all the *T. vaginalis*isolates tested, the iron limitation caused by treatment with chelators was able to regulate ADA transcriptional expression and, surprisingly, promote increased levels of mRNA. The concept that the amount of mRNA must be proportional to the enzyme activity has been put to the test with the advancements published in innovative studies, particularly in the proteomic and DNA sequencing fields of research. Current data demonstrate that approximately 40% of the amount of protein produced is proportional to the values found in mRNA. However, in the remaining 60%, the mRNA undergoes post-transcriptional regulation, which includes (i) translational interference by RNA-binding proteins or interference RNA or (ii) degradation of translated protein by other proteins (Vogel & Marcotte 2012). Studies in fungus and bacteria show that levels of mRNA and protein suffered post-transcriptional regulation in response to disturbances observed in situations of oxidative and osmolarity stress (Lee et al. 2011, Vogel et al. 2011). The lack of iron in the culture medium caused a stress condition in *T. vaginalis*, explaining the relationship between the transcription/activity of ADA enzyme.

Furthermore, several authors have reported that a lack or excess of iron may modulate the gene expression of adhesins, immunogenic proteins, protein and metabolic hydrogenosomal enzymes and cysteine proteinases in *T. vaginalis*(Torres-Romero & Arroyo 2009). A proteomic study on *T. vaginalis *trophozoites cultured in the presence or absence of iron showed changes in proteome profiles as a function of iron concentration. Mass spectrometry detected 600-640 and 540-570 spots in gels with protein samples from *T. vaginalis *grown in high-iron and low-iron conditions, respectively. Of the proteins expressed by the parasites cultured in low-iron medium, 12 were up-regulated, 19 were down-regulated and 11 had their expression abolished (de Jesus et al. 2007). The decrease in ADA activity and the increase in ADA transcription in response to iron limitation reported here support the hypothesis that iron can modulate the activity of the enzymes involved in purinergic signalling because the vaginal environment undergoes constant changes and iron levels at this site are influenced by the menstrual cycle. Therefore, in these hostile situations, enzymes are dependent on precise gene expression regulation for rapid adaptation to the environment and development of the appropriate responses to ensure the survival of the parasitic protest (Figueroa-Angulo et al. 2012).

Our findings show that serum-limitation-cultured parasites did not affect ADA activity. Conversely, iron limitation decreased enzymatic activity and increased ADA gene expression, indicating a modulation by iron. Overall, this study presents important aspects of ADA in intact trophozoites and contributes to a better understanding of the role of purinergic signalling in *T. vaginalis.*
